# Caffeine impairs resection during DNA break repair by reducing the levels of nucleases Sae2 and Dna2

**DOI:** 10.1093/nar/gkv520

**Published:** 2015-05-27

**Authors:** Michael Tsabar, Vinay V. Eapen, Jennifer M. Mason, Gonen Memisoglu, David P. Waterman, Marcus J. Long, Douglas K. Bishop, James E. Haber

**Affiliations:** 1 Department of Biology and Rosenstiel Basic Medical Sciences Research Center, Brandeis University, Waltham, MA 02454, USA; 2 Department of Radiation and Cellular Oncology and Department of Molecular Genetics and Cell Biology, University of Chicago, Chicago, IL 60637, USA

## Abstract

In response to chromosomal double-strand breaks (DSBs), eukaryotic cells activate the DNA damage checkpoint, which is orchestrated by the PI3 kinase-like protein kinases ATR and ATM (Mec1 and Tel1 in budding yeast). Following DSB formation, Mec1 and Tel1 phosphorylate histone H2A on serine 129 (known as γ-H2AX). We used caffeine to inhibit the checkpoint kinases after DSB induction. We show that prolonged phosphorylation of H2A-S129 does not require continuous Mec1 and Tel1 activity. Unexpectedly, caffeine treatment impaired homologous recombination by inhibiting 5′ to 3′ end resection, independent of Mec1 and Tel1 inhibition. Caffeine treatment led to the rapid loss, by proteasomal degradation, of both Sae2, a nuclease that plays a role in early steps of resection, and Dna2, a nuclease that facilitates one of two extensive resection pathways. Sae2's instability is evident in the absence of DNA damage. A similar loss is seen when protein synthesis is inhibited by cycloheximide. Caffeine treatment had similar effects on irradiated HeLa cells, blocking the formation of RPA and Rad51 foci that depend on 5′ to 3′ resection of broken chromosome ends. Our findings provide insight toward the use of caffeine as a DNA damage-sensitizing agent in cancer cells.

## INTRODUCTION

DNA double strand breaks (DSBs) are highly deleterious events that may lead to chromosomal abnormalities, cell death and cancer. Repair of chromosome breaks occurs by several highly conserved pathways. G1 cells predominantly repair DSBs by re-joining the broken ends through nonhomologous end-joining (NHEJ) pathways (1,2). After the cells pass ‘start’ on their way to initiate S phase, the main pathway of repair shifts to homologous recombination (HR) (2–4). An initial and essential step in HR is the 5′ to 3′ resection of the dsDNA at the DSB end, which leaves 3′ single-stranded DNA (ssDNA) tails. Both *in vivo* and *in vitro* evidence suggests that resection is initiated by the Mre11-Rad50-Xrs2 complex (MRX) together with Sae2, the budding yeast homolog of CtIP (5–8). Recently, Sae2 has been shown *in vitro* to facilitate 5′ to 3′ resection by promoting the endonuclease activity of Mre11 (9), although Sae2 itself has been suggested to have nuclease activity (10). More extensive resection depends on two separate nuclease activities, one involving Exo1 and another involving a complex containing Dna2, Sgs1, Top3 and Rmi1 (6,7,11,12). The ssDNA tail created by resection is first coated by replication protein A (RPA) that interacts with Rad52 to facilitate the formation of a filament of the Rad51 recombination protein (13–15). The Rad51 filament catalyzes a search throughout the genome for sequences homologous to the ssDNA within the filament and promotes strand invasion between the ssDNA and homologous double-stranded DNA (dsDNA). Strand invasion is followed by the initiation of DNA synthesis from the 3′ end of the invading strand and eventual repair of the DSB (16,17). When the DSB occurs in sequences that share homology on both ends of the break with a template sequence (a sister chromatid, a homologous chromosome or an ectopic donor) repair occurs by gene conversion (GC). If only one end of the DSB is capable of pairing with homologous sequences, repair proceeds by a recombination-dependent process termed break-induced replication (BIR) (18,19). Repair can also occur in a Rad51-independent fashion by single-strand annealing (SSA) when there are homologous sequences flanking a DSB (20).

In order to allow sufficient time for repair, and to prevent mitosis in the presence of a broken chromosome, cells activate the DNA damage checkpoint. Two checkpoint PI3 kinase-like protein kinases, ATM and ATR (Tel1 and Mec1 in yeast, respectively), are recruited to the DSB and phosphorylate a cascade of downstream effectors that, in turn, prevent the cells from dividing until the damage is repaired (21–24). In budding yeast, the scaffolding protein Rad9 is recruited to the DSB, where it is phosphorylated by Mec1 (24). Rad9 then mediates the autophosphorylation of Rad53 (Chk2) and Chk1 (22,25). Rad53 phosphorylates and inhibits Cdc20, an activator of the anaphase-promoting complex. This inhibition, along with activation of Chk1, stabilizes Pds1 (securin) and prevents mitosis (22,26). After repair is complete, the DNA damage checkpoint is turned off to allow the cells to resume cell cycle progression, a process termed recovery. If the damage cannot be repaired, the cells can eventually turn off the checkpoint by a process termed adaptation (27,28).

Another target of Mec1 and Tel1 kinase activity is serine 129 of histone H2A. This modification, termed γ-H2AX, is evolutionarily conserved; ATM and ATR rapidly phosphorylate mammalian H2AX-S139 in response to DNA damage (29–32). The modification spreads as far as 100 kb around the DSB in yeast cells, and 1 Mb around a DSB in mammalian cells, and serves to recruit repair factors to the vicinity of the DSB (29,31,33). Cells that lack the ability to phosphorylate H2A-S129 (H2A-S129A) adapt faster than WT cells, suggesting this modification plays a role in determining the length of arrest (34,35). Surprisingly, cells expressing histone H2A-S129A have a rate of 5′ to 3′ resection of the DSB ends greater than that in WT cells (6.8 kb/h versus 4 kb/h), which implies that having more ssDNA or more resection byproducts does not, in and of itself, signal for checkpoint maintenance (36,37). γ-H2AX in yeast is rapidly removed from chromatin after repair by a yet unknown factor and then dephosphorylated by the PP4 phosphatase complex (38).

Here we asked if Mec1 and Tel1 are constitutively required to maintain γ-H2AX after its formation. We used caffeine to inhibit Mec1 and Tel1 after the formation of γ-H2AX. Caffeine acts as a PI3K inhibitor and has been shown to inhibit ATM and ATR in mammalian cells, *Schizosacchromyces pombe* and *Saccharomyces cerevisiae* (39–45). We find that γ-H2AX is retained around a DSB after caffeine treatment indicating that the modification is stable, and does not depend on continuous Mec1/Tel1 activity.

In mammalian cells, the tumor suppressor p53 becomes active following DNA damage in the G1 phase, and maintains a robust G1 checkpoint (46). In contrast, the S phase and G2/M checkpoints are completely dependent on ATM and ATR, and do not require p53 (46). Indeed, caffeine treatment was shown to sensitize p53-deficient tumor cells to ionizing irradiation. This effect was hypothesized to occur due to the caffeine-mediated inhibition of the G2/M checkpoint (47–49). However, caffeine could not further radiosensitize both mammalian and DT-40 cells that are deficient for HR, suggesting that the mechanism of sensitization involves inhibition of HR rather than checkpoint control (50–52). A recent study showed that caffeine treatment interferes with mammalian Rad51-mediated joint molecule formation *in vitro* as well as gene targeting *in vivo* (53). Here we show that caffeine treatment impairs resection in budding yeast and HeLa cells. We show in budding yeast that inhibition of homologous recombination occurs independently of inhibition of the DNA damage checkpoint, by interfering with the first step in repair: resection of the DSB ends. The impairment of resection correlates with a reduction in the abundance of both Sae2 and Dna2 following caffeine treatment. We show that both nucleases are targeted for proteasomal degradation. In particular, we show that Sae2 is an unstable protein, as it is degraded rapidly following caffeine or cycloheximide (CHX) treatment even in the absence of DNA damage. Taken together, these data suggest that caffeine treatment targets one of the earliest steps in HR, independent of its inhibition of ATM/ATR.

## MATERIALS AND METHODS

### Yeast strains and plasmids

All yeast strains used in this study are described in Table 1. Deletions of open reading frames (ORFs) were carried out using single-step-PCR-mediated transformation of yeast colonies (54) or introduced by genetic crosses. Primer sequences are available on request.

**Table 1.** Strain list

Strain name	Genotype
JKM179	*hoΔ hml::ADE1 MATα hmr::ADE1 ade1-110 leu2,3-112 lys5 trp1::hisG ura3-52 ade3::GAL:HO*
JKM139	*JKM179 isogenic, MATa*
YML002	*JKM139 (HO cut site deleted) Cen3HOcs::HPH 2 kb homology to the left of the HOcs inserted to the right of Cen3, 97700-97800 Ch6::HOcs-NAT*
tGI354	*JKM139 MATa-inc (+CA), arg5,6::MATa-HPH*
R1304 (G. Ira)	*JKM139 DNA2-9xMYC::TRP1*
R1306 (G. Ira)	*JKM139 Fun30-3x-FLAG::KAN EXO1-9x MYC::TRP1*
R1307 (G. Ira)	*JKM179 Fun30-13x Myc::NAT RFA1-3X FLAG::KAN*
R1206 (M. Foiani)	*JKM139 Mre11-Myc::KAN SAE2-6PK::TRP1 Exo1-Flag::KANMX*
CY9666 (M. Foiani)	*JKM139, Sae2-6PK, atg1::KAN*
VE18	*JKM179, GFP-ATG8 URA3 (pRS316)*
VE150	*R1304 atg1::URA3*
VE114	*JKM179 GFP-Atg8 URA3 (pRS316) atg1::KAN*
sDW133	*JKM179 pdr5::HPH rpn4::NAT SAE2-6PK::TRP1*
sDW134	*JKM179 pdr5::HPH rpn4::NAT DNA2-9xMYC::TRP1*

### ChIP

Chromatin immunoprecipitation (ChIP) was carried out as described in (29). α-γH2AX purchased from Abcam (15083).

### Western blotting

Western blotting in *S. cerevisiae* was carried out using the trichloroacetic acid (TCA) protocol described by Pellicioli *et al*. (55). α-Rho1 antibody was a generous gift from Satoshi Yoshida (Brandeis, MA, USA). α-GFP (ab290), α-CMyc (ab32) and α-PGK1 (ab113687) antibodies were obtained from Abcam. α-V5 antibody was obtained from Life Technologies. HeLa cells were harvested in lysis buffer containing 1% NP-40, 0.1% SDS, 0.5% sodium-deoxycholate, 1× protease inhibitor cocktail (Roche). Western blotting was performed using antibodies against human CTIP (kind gift of Richard Baer) and α-Tubulin (Ab-2; Fitzgerald). Signal intensity was measured in Image J. CTIP levels were determined by normalizing signal to Tubulin signal intensity.

### qPCR resection assay

qPCR resection assay was carried out as previously described (36). In short, qPCR was used to detect product at varying distances from the DSB. The signal was then normalized to qPCR signal at an uninvolved site (either *PHO5* or *ARG5,6*) and to the signal present before a DSB was induced (0 h). Resection rates were calculated by examining each curve on the plot of distance versus PCR signal and determining the time and distance when the PCR value reached 0.75 (calculated by interpolating between the nearest data points).

### Southern blotting

Southern blot analysis was performed as previously described (36). Genomic DNA was purified and digested with the appropriate restriction enzyme (*Pvu*I for YML002, *EcoR*I for all other Southerns). YML002 probe detected the homologous sequences. All other strains were probed with an *MAT* distal probe (56). For tGI354 derivatives, the donor signal served as a loading control. Bands were normalized to the loading control and then to the 0 h. JKM179 was cut with *Sty*I prior to hybridization with the *MAT* distal probe, after which the membrane was stripped and reprobed with *ACT1* probe as a loading control.

### Mammalian cell culture conditions

HeLa cells were grown in DMEM media (Invitrogen) supplemented with 10% fetal bovine serum, and 1× penicillin/streptomycin (Invitrogen). Cells were cultured at 37°C with 5% CO_2_.

### Immunofluorescence

HeLa cells (1.0 × 10^5^) were plated in 6-well dishes (Corning) containing a 22 × 22 glass coverslip (Fisherbrand) and allowed to adhere overnight. For Rad51 and RPA focus formation, caffeine was added at the indicated doses 1 h prior to irradiation with 6 Gy using a Maxitron generator and allowed to recover for 4 h prior to fixation. Cytosolic proteins were pre-extracted with HEPES/TritonX-100 buffer (20 mM HEPES pH 7.4, 0.5% Triton X-100, 50 mM NaCL, 3 mM MgCl_2_, 300 mM sucrose in ddH_2_O) at room temperature for 10 min. Cells were subsequently fixed with 3% paraformaledhyde, 3.4% sucrose in PBS for 10 min at room temperature. Cells were blocked in 1% BSA in PBS for 15 min and stained with human RAD51 serum (kind gift from Akira Shinohara, Dilution 1:1500) and RPA antibodies (Calbiochem (NA18), Dilution 1:2000) overnight at 4°C. Cells were washed with PBS and stained with Alexa Fluor conjugated secondary antibodies (Invitrogen, Dilution 1:1000) for 1 h at room temperature. Cells were washed with PBS and allowed to air dry. Coverslips were mounted on slides with vectashield containing 4’,6-diamidino-2-phenylindole (DAPI). To image cells, 1 micron sections were acquired on a scanning laser confocal microscope (LSM510, Zeiss) using LSM software. Images were acquired with a 60× objective. Z stacks were compressed into a single image and analyzed using ImageJ software (NIH). The number of RAD51 and RPA foci in at least 75 random nuclei from three independent experiments were counted. Statistical significance was determined using the Wilcoxon rank sum test.

## RESULTS

### Loss of γ-H2AX after DSB repair occurs in a locus-autonomous fashion

In yeast, histone H2A is phosphorylated by Mec1 and Tel1 in response to DNA damage (29). Following repair, γ-H2AX is removed from the surrounding DNA and then dephosphorylated by the phosphatase Pph3 (38). When the damage cannot be repaired, γ-H2AX is maintained around the DSB, apparently signaling the persistence of damage (40). We first asked if γ-H2AX is removed from the chromatin surrounding a repairable DSB even when an irreparable DSB is present in the same nucleus.

**Figure 1.**
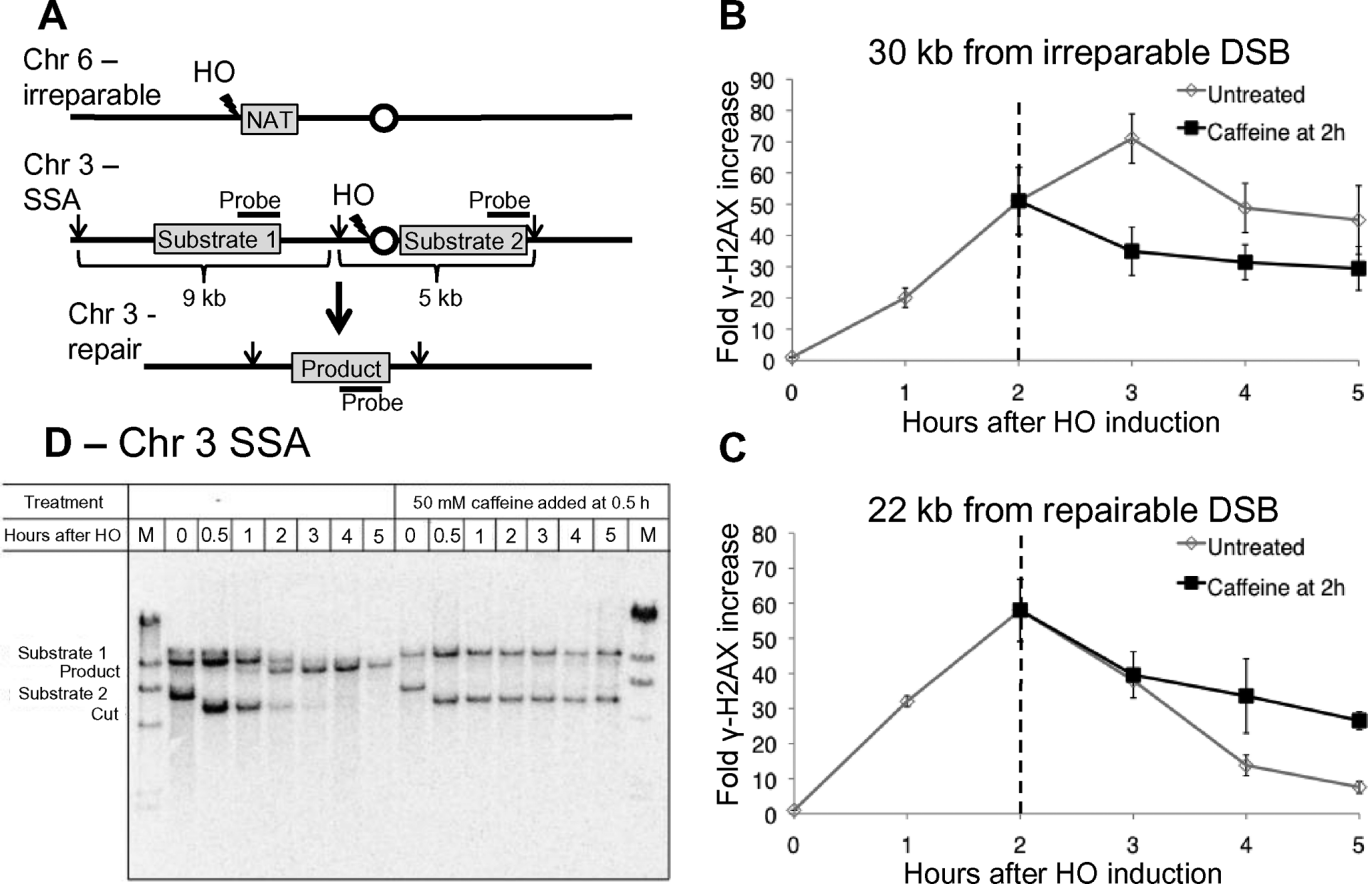
γH2AX is stable following caffeine treatment. **(A)** Schematic of YML002 strain. In a strain lacking *MAT, HML* and *HMR*, an irreparable DSB is induced on Chr 6, and a second DSB surrounded by flanking homologies is created on Chr 3. Repair by SSA leads to loss of the centromere. Arrows indicate *Pvu*I restriction sites. **(B)** ChIP signal for γ-H2AX 30 kb from the irreparable DSB. Fifty micromolar caffeine added to the culture 2 h following HO induction (black). Dashed line marks the time of caffeine addition. **(C)** ChIP signal for γ-H2AX 22 kb from repairable DSB (bottom graph). Fifty micromolar caffeine added to the culture 2 h following HO induction (black). Dashed line marks the time of caffeine addition. **(D)** Repair of the SSA event on Chr 3 without treatment and when 50 mM caffeine is added 0.5 h after HO induction.

To address this question, we designed a system that contains two HO endonuclease recognition sites and a galactose-inducible HO endonuclease gene (34). The recognition site on chromosome 3 (Chr 3) is flanked on each side by identical copies of a 1-kb DNA sequence in the same orientation. Addition of galactose to the media induces HO expression and leads to formation of a DSB that is repaired by single strand annealing (SSA), creating a deletion of one of the two copies of the duplicated sequence and intervening sequences (Figure 1A). An additional HO cleavage site was introduced at the left arm of Chr 6. This cut site does not share homology elsewhere in the genome and thus is irreparable by HR (34). This strain also carries deletions of the HO cleavage site at the *MAT* locus as well as its two homologous donor sequences *HML* and *HMR*. We analyzed γ-H2AX by chromatin immunoprecipitation (ChIP) as described in Materials and Methods (Figure 1B). Repair was assayed by Southern blot hybridization (Figure 1D). γ-H2AX peaks 1–2 h following induction of both DSBs and spreads more than 30 kb on either side of the break. Phosphorylation at the irreparable DSB persists throughout the experiment (Figure 1B), but phosphorylation around the repairable DSB is removed following repair (Figure 1C). Thus, the removal of γ-H2AX is not a global event, but is triggered by the state of repair of each individual damage site.

### Prolonged phosphorylation of H2AX does not require continuous Mec1 and Tel1 activity

In the presence of persistent damage, γ-H2AX spreads in a Mec1-dependent manner to further distance along the broken chromosome as resection proceeds over many hours (40). Active transcription leads to removal of γ-H2AX from transcribed regions, but after transcription is shut off γ-H2AX is rapidly re-established in a Mec1-dependent fashion (57). Both of these observations indicate that Mec1 is active and able to phosphorylate histone H2A long after a DSB has occurred. It is not yet known, however, if the kinases are continuously required to maintain γ-H2AX after damage.

To address this question, we added 50 mM caffeine to inhibit Mec1 and Tel1 2 h after HO induction and γ-H2AX establishment. Previous studies have shown that caffeine at this concentration can efficiently inhibit the DNA damage checkpoint and prevent DNA damage induced γ-H2AX enrichment (39,40). If Mec1 and Tel1 are continuously required for the phosphorylation of H2AX around the break, the inhibition of these two kinases should cause a decrease in phosphorylation around the irreparable break. Caffeine treatment 2 h after HO induction did not result in a significant decrease in γ-H2AX signal at a distance 30 kb from the DSB site at later time points and phosphorylation persisted for at least 5 h (Figure 1B). In the untreated cells, γ-H2AX signal continued to increase until 3 h. This result indicates that caffeine treatment is effective in preventing additional phosphorylation. Inhibition of Mec1 and Tel1 by caffeine led to a slight reduction in γ-H2AX signal but this signal remains more than 30-fold higher than 0 h. Thus maintenance of γ-H2AX around an unrepaired DSB does not require continuous ATM/ATR activity.

Surprisingly, γ-H2AX around the repairable DSB also persisted up to 5 h in the presence of caffeine (Figure 1C). This observation raised the possibility that caffeine treatment prevents SSA-dependent repair of the break.

### Caffeine treatment inhibits single-strand annealing by impairing resection

To see if caffeine treatment affects repair, we monitored the kinetics of repair of the SSA event in the strain described above by a Southern blot (Figure 1D). Samples were taken at intervals after HO induction, with or without caffeine treatment. In the untreated cells, repair product appeared 1 h after HO induction, and by 5 h the bands representing the original two flanking sequences were lost and only the product band was present, indicating complete repair. When the cells were treated with 50 mM caffeine 30 min after HO induction, repair was completely inhibited. We then tested if lower concentration of caffeine treatment with 10 and 20 mM caffeine 30 min after HO induction also led to a notable reduction in product appearance 5 h after HO induction (Supplementary Figure S1A and S1B). Repair was strongly inhibited by 20 mM treatment and delayed with addition of 10 mM. Notably, caffeine treatment led to the persistence of the 5 kb cut fragment formed after formation of the DSB at all concentrations (Figure 1D and Supplementary Figure S1A). In the untreated cells, this band appeared after DSB formation and disappeared in a resection-dependent manner (11). These observations suggest that caffeine treatment impairs resection.

To examine resection more directly, we used strain JKM179 in which an HO-induced DSB at the *MAT* locus cannot be repaired because both *HML*α and *HMR***a** donors have been deleted. We monitored the persistence of the 700 bp StyI fragment created by HO cleavage at *MAT*. Treatment of cells with 50 mM caffeine 30 min after HO induction had no significant effect on resection of the region immediately adjacent to the DSB (Supplementary Figure S1C and S1D). Moreover, repair by NHEJ, a repair pathway that competes with HR and is preferred when the DSB ends are not resected (3,58), was not elevated following caffeine treatment compared to untreated cells (Supplementary Figure S1E) suggesting that the ends of the DSB are resected sufficiently to interfere with this pathway.

**Figure 2.**
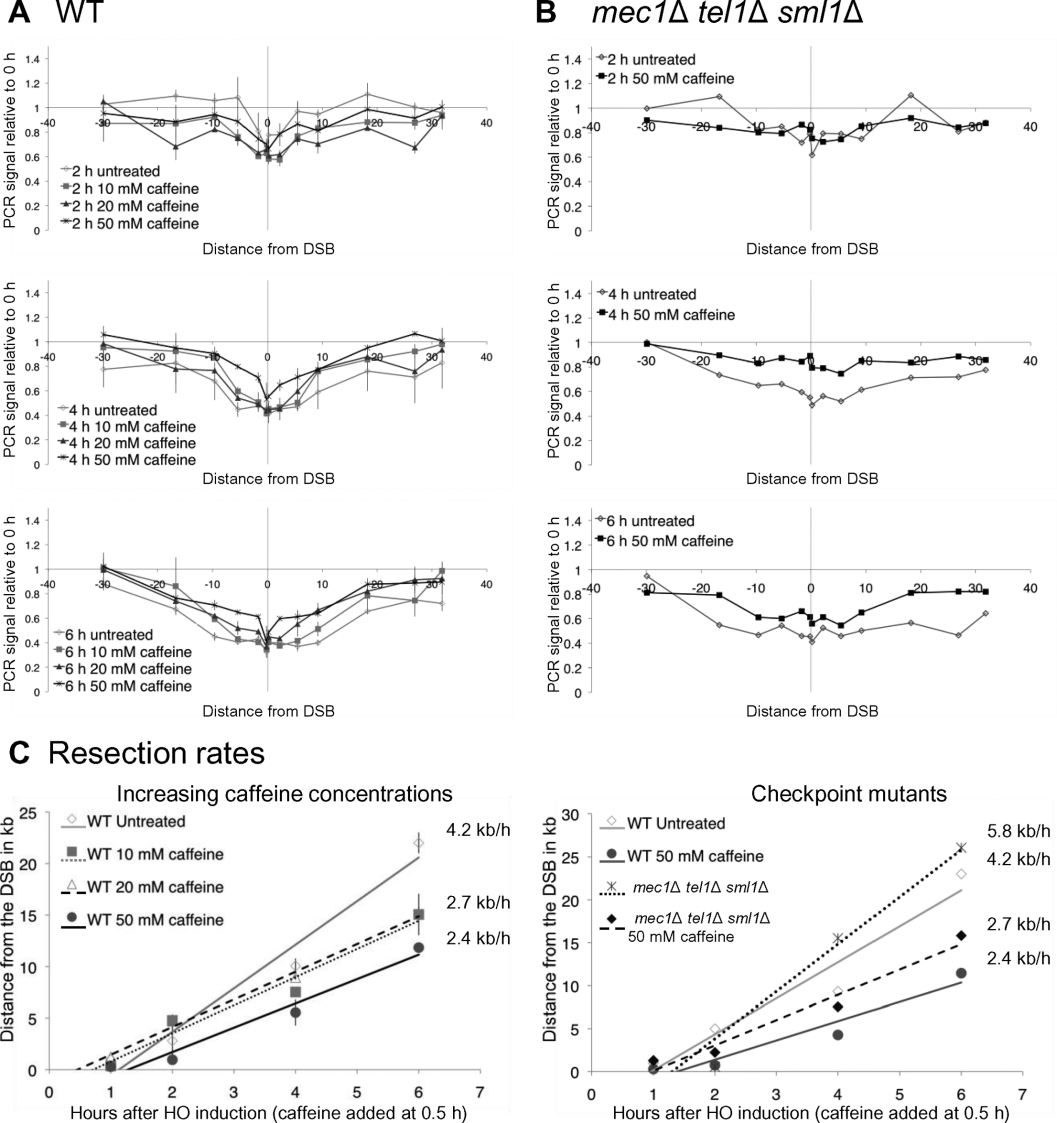
Caffeine treatment impairs resection independent of Mec1 and Tel1. Cells were arrested in G2/M by nocodazole treatment 3 h prior to HO induction. **(A)** Resection in strain JKM179 without caffeine treatment (diamonds), or when 10 mM caffeine (squares), 20 mM caffeine (triangles) or 50 mM caffeine (crosses) was added 0.5 h after HO induction. Error bars represent ranges. **(B)** Resection in JKM179 *mec1*Δ *tel1*Δ *sml1*Δ without caffeine treatment (gray) and when 50 mM caffeine was added 0.5 h after HO induction (black) at different times after HO induction. **(C)** Rates were calculated by determining the time and distance when the PCR signal fell to 75% of signal at 0 h (36). These values were plotted on time-versus-distance graphs, and the rates were determined by linear regression analysis. Left graph represents resection measurements seen in (A) error bars represent the range. Right graph represents resection measurements seen in (B).

In the donorless strain described above, resection can be monitored by a quantitative PCR (qPCR) assay described previously (36). Resection depends on Cdk1 activation and is inhibited in G1-arrested cells (3,4). Because caffeine might impair resection by allowing cells to evade the DNA damage checkpoint and progress to the next G1, we arrested the cells in the G2/M phase of the cell cycle by adding nocodazole 3 h prior to HO induction. At that point, 95% of the cells were at the dumbbell stage characteristic of G2/M arrest. In untreated cells, the resection rate was 4.2 kb/h (Figure 2A and C), which is comparable to the previously published rate of 4 kb/h (36) determined by this method. Fifty micromolar caffeine treatment of nocodazole-arrested cells slowed down resection to 2.4 kb/h (Figure 2A and C). This effect was seen even in lower concentrations of caffeine (10 and 20 mM) although the rate of resection measured at lower concentrations was slightly higher than that seen at 50 mM caffeine (2.7 kb/h at 10 and 20 mM) (Figure 2A and C). Thus, although resection very close to the DSB does not seem to be affected, resection is indeed significantly impaired further from the DSB. This result indicates that caffeine impairs resection directly, rather than by allowing the cells to progress through the cell cycle.

Next we monitored resection in a *mec1*Δ *tel1*Δ *sml1*Δ donorless strain to see if caffeine impairs resection by inhibiting Mec1 and Tel1. The deletion of *SML1* is required to allow viability in the absence of *MEC1*. In meiosis Mec1 and Tel1-dependent phosphorylation is required for Sae2's function (59); this modification might be important for resection; however in mitosis deletion of *MEC1* leads to accelerated resection (60). To allow cells to remain in G2/M following damage, we arrested cells in nocodazole 3 h prior to HO induction. The resection rate in a *mec1*Δ *tel1*Δ *sml1*Δ donorless strain was higher than WT (5.8 kb/h) (Figure 2B and C), most likely due to the inability of these cells to phosphorylate histone H2A (36,37,60). Fifty micromolar caffeine treatment in this *mec1*Δ *tel1*Δ *sml1*Δ strain also impaired resection (2.7 kb/h). We conclude that caffeine slows down resection independently of checkpoint kinase inhibition.

### Caffeine treatment leads to degradation of resection enzymes

Caffeine could impair resection by directly blocking the function of resection enzymes or by inhibiting their post-translational modifications, or by an indirect mechanism such as inhibition of protein synthesis. Cdk1-dependent phosphorylation of Dna2 and Sae2 has been shown to be important for resection (61–63), whereas both Mre11 and Exo1 have been shown to be phosphorylated by the checkpoint kinases (63,64). Moreover, a recent study showed that treatment of yeast with the TORC1 inhibitor rapamycin or the histone deacetylase inhibitor valproic acid (VPA) led to the degradation of Sae2 and Exo1 (65).

**Figure 3.**
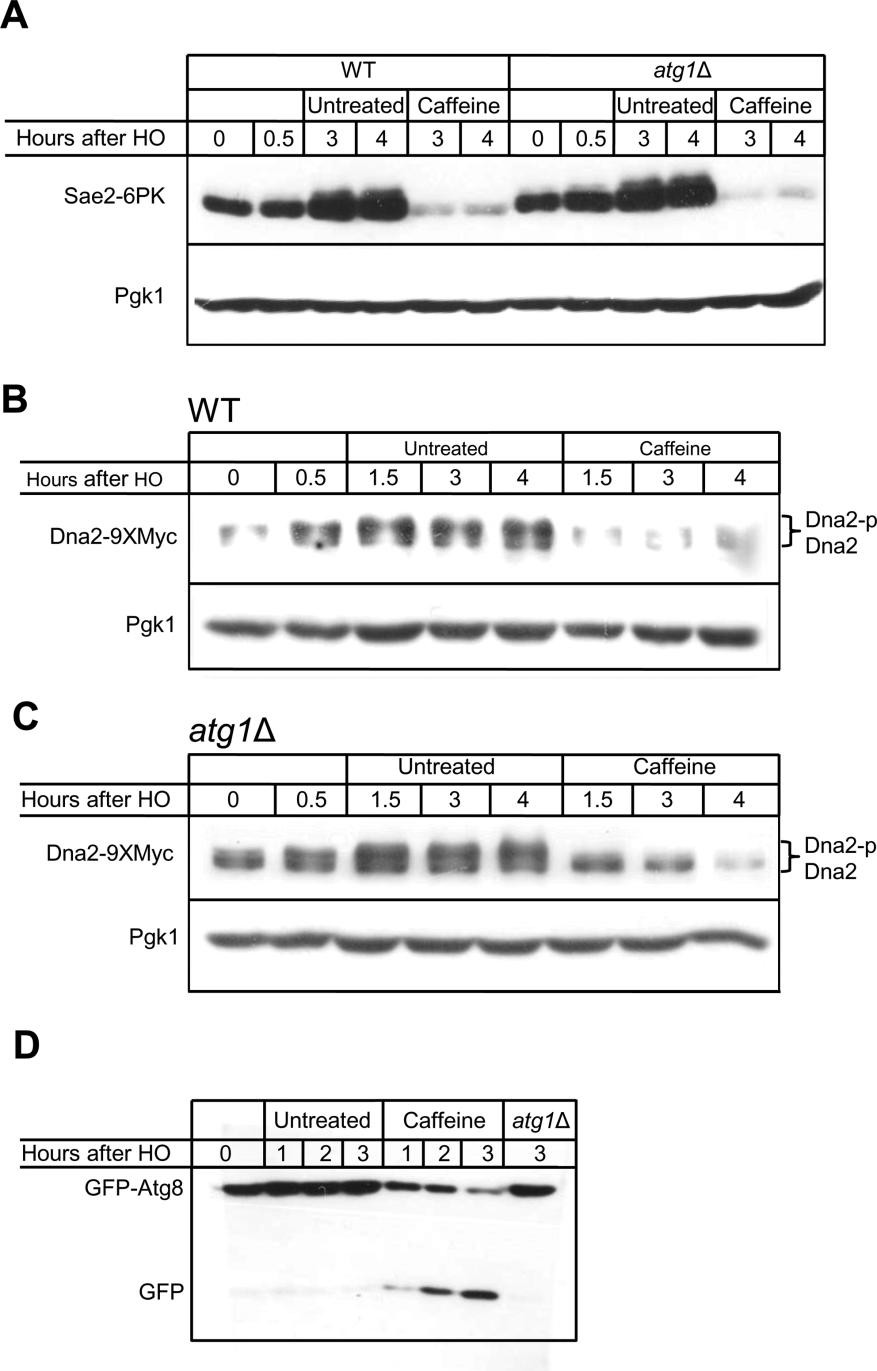
Caffeine treatment leads to degradation of resection enzymes. **(A)** Caffeine leads to degradation of Sae2. WT cells (left six lanes) and *atg1*Δ (right six lanes) were arrested in nocodazole prior to HO induction, and treated with 20 mM caffeine 0.5 h following HO induction. **(B)** Caffeine leads to degradation of Dna2. Cells were arrested in nocodazole prior to HO induction, and treated with 20 mM caffeine 0.5 h following HO induction. **(C)** Deletion of *ATG1* does not prevent Dna2 degradation after caffeine treatment. Twenty micromolar caffeine was added to the media 0.5 h after HO induction in *atg1*Δ cells. **(D)** Caffeine treatment induces autophagy. A GFP band released from GFP-Atg8 appears 2 h after 10 mM caffeine addition.

We assayed the abundance of the resection enzymes using western blots. We first arrested cells with nocodazole prior to HO induction and treated the cells with 20 mM caffeine 30 min after HO induction of an irreparable DSB. 20 mM caffeine was used as this concentration showed a more severe repair phenotype by SSA than 10 mM (Supplementary Figure S1A and S1B). Similar to VPA treatment, treatment with 20 mM caffeine markedly reduced Sae2 levels ≥ 3 h after formation of a DSB (Figure 3A and Supplementary Figures S2A and S3A). Sae2 levels were reduced 3-fold compared to the level prior to HO induction and 6-fold compared to untreated cells (Figure 3A and Supplementary Figure S3A).

Phosphorylation of Dna2 by Cdk1 is required for its recruitment to the DSB and subsequent Mec1-dependent phosphorylation (61). Dna2 appears as two bands at 0 h in G2/M arrested cells, the upper band most likely due to the Cdk1 phosphorylation. After DSB formation Dna2 increases in abundance and appears to migrate higher on the gel, probably a result of Mec1-dependent phosphorylation (Figure 3B). Caffeine treatment 0.5 h after DSB formation also reduced Dna2 levels 5-fold compared to untreated cells when compared 4 h after DSB induction (Figure 3B and Supplementary Figure S3C). After caffeine treatment the higher mobility band disappeared, while the lower, presumably unphosphorylated, band persisted, albeit at lower levels than prior to DSB formation (Figure 3B).

Unlike VPA treatment (65), caffeine treatment did not significantly alter the abundance of Exo1 (Supplementary Figure S2C). Mre11 levels were slightly reduced following caffeine treatment compared to untreated samples (Supplementary Figure S2A), but remained at the levels detected at G2/M. Levels of Fun30, a chromatin remodeler recently shown to promote resection (36,37), were not affected by either caffeine or VPA treatments (Supplementary Figure S2B).

Both VPA and rapamycin have been suggested to cause Sae2 degradation by induction of autophagy (65), and VPA was also shown to decrease the rate of resection of DSB ends. Because rapamycin treatment induces autophagy through inhibition of TORC1 signaling, and caffeine is also a TORC1 inhibitor (66), we hypothesized that caffeine treatment might induce autophagy in yeast, thereby impairing resection. We note that caffeine has been shown to stimulate autophagy in mammalian cells and in the yeast *Zygosaccharomyces bailii* (66–69).

A commonly used technique for monitoring autophagy is the cleavage of GFP-Atg8 by vacuolar proteases (70). After the induction of autophagy, the ubiquitin-like protein Atg8 is lipidated and incorporated into autophagosomes. Subsequently, Atg8-containing autophagosomes are transported to the vacuole, where Atg8 is degraded. Tagging Atg8 at the N-terminus with GFP allows monitoring this process by western blotting or fluorescence microscopy. Although GFP-Atg8 is degraded in the vacuole by proteases, the relative resistance of GFP to proteolytic cleavage results in the appearance of a free GFP band upon autophagy induction (70). Treatment of cells with 10 mM caffeine led to the appearance of a free GFP band 1 h after caffeine treatment (Figure 3D). This result is comparable to other autophagy-inducing agents such as rapamycin (71) and VPA (65) and demonstrates that caffeine induces autophagy in *S. cerevisiae*.

Sae2 and Dna2 protein levels were markedly lower after treatment with caffeine. Because deletion of *ATG1*, a protein kinase required for autophagy (72), was reported to partially rescue of Sae2 degradation after VPA treatment (65), we hypothesized that the same might be true for caffeine. However, caffeine treatment resulted in loss of Sae2 even in *atg1*Δ cells (Figure 3A and Supplementary Figure S3B). Likewise, treatment of *atg1*Δ cells with rapamycin resulted in loss of Sae2 (Supplementary Figure S4A). Because deletion of Atg1 did not rescue Sae2 from degradation following either rapamycin or caffeine treatments, we attempted to replicate the rescue of Sae2 following VPA treatment (65). In contrast to the reported result in Roberts *et al*., we find the deletion of *ATG1* failed to rescue Sae2 from VPA-mediated degradation (Supplementary Figure S4B).

We also investigated the localization of Sae2 by integrating a C-terminal mCherry tag at the endogenous *SAE2* gene. Sae2 is predominantly nuclear localized (Supplementary Figure S4C). As an alternative way to induce autophagy in cells with HO-induced DNA damage, we overexpressed a dominant-active form of the autophagy protein Atg13 (Atg13–8SA), which induces autophagy independently of TORC1 inhibition and without impairing the cell cycle (73). Induction of autophagy has been shown to cause cytoplasmic mislocalization of the nuclear protein Pds1 (74); however, even in cells where Atg13–8SA is overexpressed, Sae2 remains localized to the nucleus and its fluorescence intensity is unchanged (Supplementary Figure S4C). Taken together, these results show that the instability of Sae2 is not predominantly dependent on autophagy.

Deletion of *ATG1* also failed to prevent the degradation of Dna2 following caffeine treatment, as Dna2 was almost fully degraded by 2.5 h after caffeine treatment (3 h following HO induction) (Figure 3C and Supplementary Figure S3D). As in the WT strains, after caffeine treatment the higher migrating Dna2 was rapidly lost, but the lower migrating forms persisted longer. These data suggest that a pathway other than autophagy, most likely proteasomal degradation, mediates most of the degradation of both Sae2 and Dna2 in response to caffeine treatment.

### Sae2 and Dna2 are targets of proteasomal degradation

**Figure 4.**
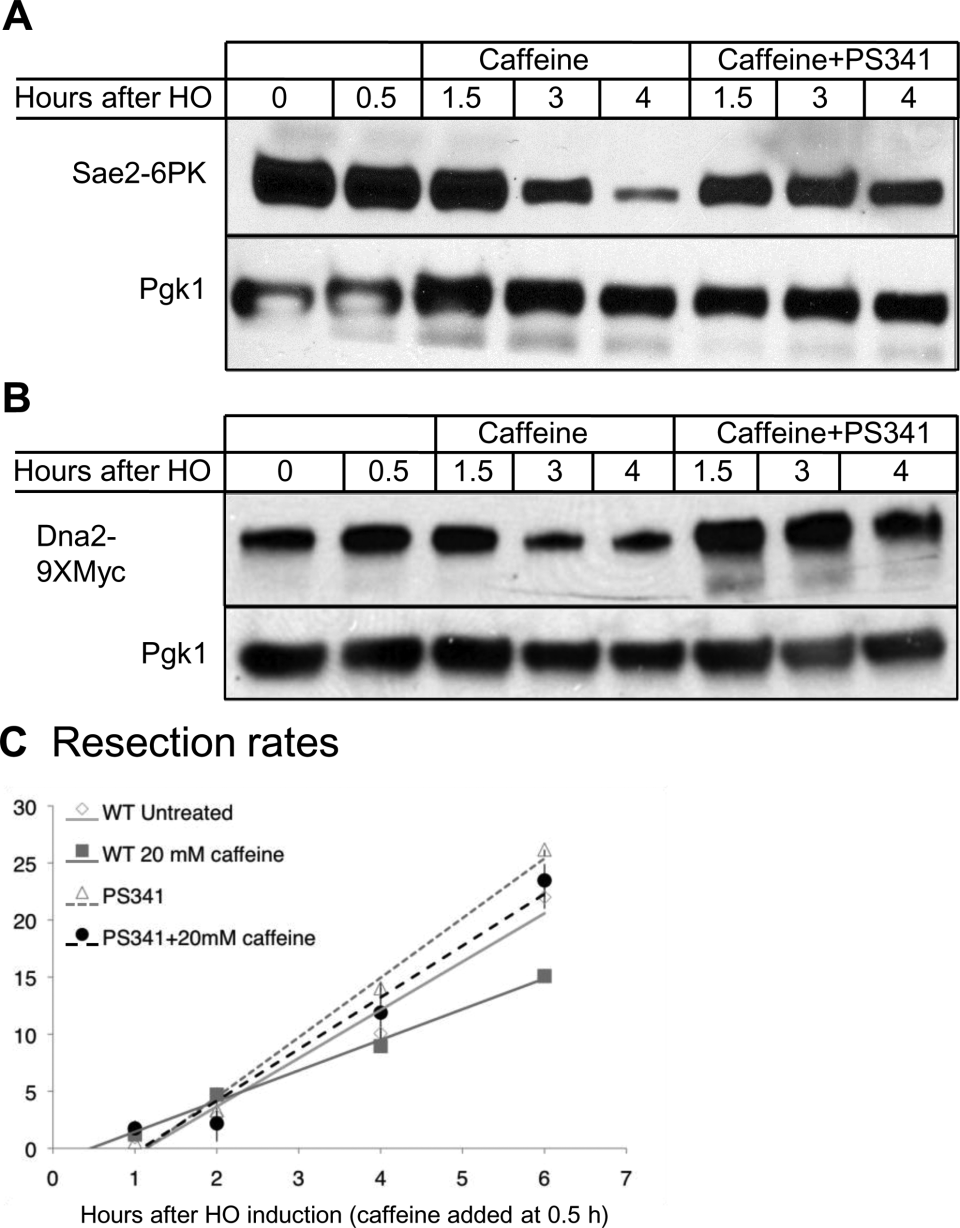
Sae2 and Dna2 are targets of proteasomal degradation. **(A)** Western blot to detect Sae2 levels in *rpn4*Δ *pdr5*Δ cells after caffeine treatment. HO was induced in G2/M-arrested cells. Forty five minutes after HO induction, cells were treated with 100 mM PS-341, and 55 min after HO induction 20 mM caffeine was added to the medium. **(B)** Western blot to detect Dna2 levels in *rpn4*Δ *pdr5*Δ cells after caffeine treatment. HO was induced in G2/- arrested cells. Forty five minutes after HO induction, cells were treated with 100 mM PS-341, and 55 min after HO induction, 20 mM caffeine was added to the medium. **(C)** Rate of resection after inhibition of proteasomal degradation. Cells were arrested in G2/M by nocodazole treatment for 3 h. HO was induced at 0 h. Resection was measured in JKM179 (WT) or *rpn4*Δ *pdr5*Δ (PS341). Following addition of 100 mM PS-341 at the time of HO induction, 20 mM caffeine was added at 0.5 h. Rates were calculated by determining the time and distance when the PCR signal fell to 75% of signal at 0 h (WT rates are as shown in Figure 2A, PS341 rates calculated from graphs shown in Supplementary Figure S5) (36). These values were plotted on time-versus-distance graphs, and the rates were determined by linear regression analysis. Error bars represent the range.

To determine if Dna2 and Sae2 are substrates of proteasomal degradation following caffeine treatment, we repeated the time-courses in the presence of proteasome inhibitor PS-341 (75). In order to sensitize cells to the inhibitor, we deleted *PDR5*, a drug transporter and *RPN4*, a transcriptional regulator of proteasome genes (75–77). After nocodazole arrest, HO was induced, and 45 min later cells were treated with 100 μM of PS-341. Ten min after PS-341 addition (55 min after HO induction), 20 mM caffeine was added to the medium. Treatment with PS-341 prior to caffeine treatment stabilized Sae2 levels when compared to cells not treated with the proteasome inhibitor (Figure 4A). Dna2 was partially stabilized in *pdr5*Δ *rpn4*Δ even without addition of PS-341 (compare Figure 4B with Figure 3B), but completely stabilized when the cells were treated with PS-341 prior to caffeine treatment. Such stabilization in *rpn4*Δ even without PS-341 addition has been shown for other proteasomal targets (76). These data suggest both Sae2 and Dna2 are degraded by the proteasome following caffeine treatment.

We then tested if inhibition of proteasomal degradation would prevent the reduction of resection seen after caffeine treatment. We arrested *pdr5*Δ *rpn4*Δ cells in G2/M by nocodazole treatment for 3 h, after which HO was induced and 100 μM PS-341 added to the media. The rate of resection seen after inhibition of proteasomal degradation in this strain was comparable to WT (Figure 4C and Supplementary Figure S5). After inhibition of proteasomal degradation, treatment with 20 mM caffeine at 0.5 h failed to impair resection (Figure 4C and Supplementary Figure S5), strongly suggesting that the proteasomal degradation of Sae2 and Dna2 following caffeine treatment is the main pathway by which caffeine treatment impairs resection.

### Sae2 has a short half life that is not dependent on the DNA damage checkpoint

**Figure 5.**
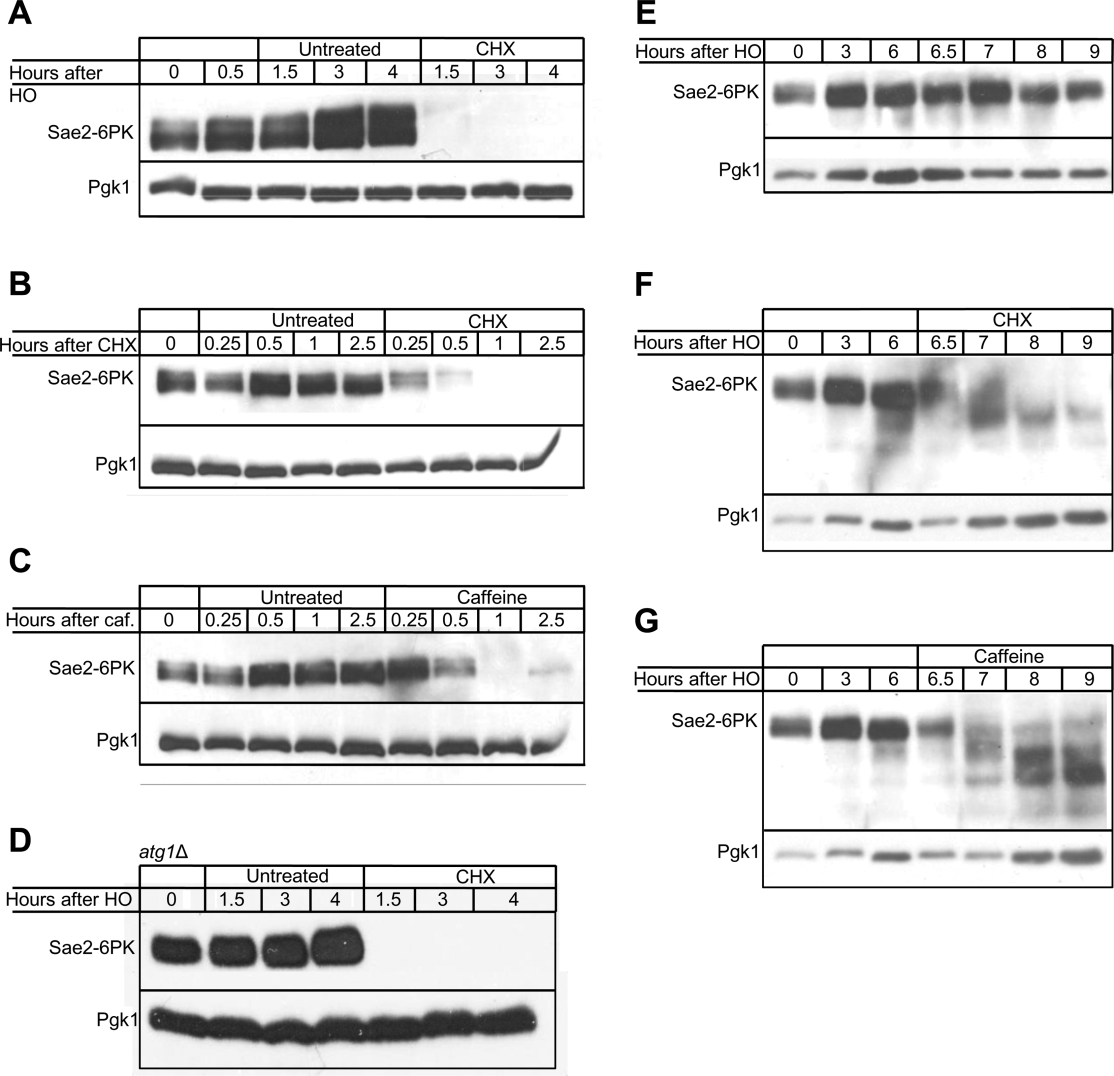
Sae2 has a short half-life regardless of DNA damage. **(A)** The stability of Sae2 was assessed after induction of a DSB. Cells were arrested in nocodazole prior to HO induction and treated with 100 μg/ml CHX 0.5 h after HO induction. **(B)** The stability of Sae2 was assessed without induction of DSB. Cycling cells were treated with 100 μg/ml CHX. **(C)** The stability of Sae2 was assessed without induction of DSB. Cycling cells were treated with 20 mM caffeine. **(D)** The degradation of Sae2 following CHX treatment was assayed in an *atg1*Δ strain. Cells were arrested in nocodazole prior to HO induction and treated with 100 μg/ml CHX 0.5 h after HO induction. **(E)** Sae2 is stable up to 9 h following DNA damage. Sae2 was monitored for 9 h following HO induction. **(F)** Sae2 is degraded after DNA damage dependent induction. Cells were treated with 100 μg/ml CHX 6 h following HO induction. **(G)** Sae2 is degraded after DNA damage dependent induction. Cells were treated with 20 mM caffeine 6 h following HO induction.

TORC1 regulates protein synthesis by promoting translation initiation, assembly of the translation machinery and regulating mRNA turnover (78). Therefore, inhibition of TORC1 by rapamycin leads to protein synthesis inhibition in both yeast and mammals (79–81). Because caffeine also inhibits TORC1, we hypothesized that the loss of Sae2 could be the result of inhibiting protein synthesis. We therefore tested if Sae2 is lost after inhibiting protein synthesis by CHX. Cells were arrested in G2/M by nocodazole, after which HO was induced. Thirty minutes following HO induction, cells were treated with 100 μg/ml CHX. CHX treatment resulted in complete loss of Sae2 1 h after treatment (Figure 5A).

A recent study suggested that Sae2 is degraded following DNA damage in both autophagy- and proteasome-dependent pathways (62). We therefore tested if the rapid loss of Sae2 following treatment with CHX is DNA-damage dependent. We treated cycling cells with 100 μg/ml CHX or 20 mM caffeine. Treatment with CHX resulted in loss of Sae2 as soon as 30 min after treatment (Figure 5B). Caffeine treatment led to a complete loss of Sae2 one h after treatment (Figure 5C). These results demonstrate that Sae2 exhibits a short half-life independent of DNA damage. Furthermore, these results suggest that the instability of Sae2 is not cell cycle dependent.

The effect of CHX on autophagy is controversial. While some studies suggest that CHX inhibits starvation-induced autophagy in both yeast and mammals (82,83), others show that CHX cannot inhibit rapamycin-induced autophagy (84). To test if treatment of yeast with CHX leads to loss of Sae2 by stimulating autophagy, we performed a western blot for Sae2 in an *atg1*Δ strain. As with caffeine, we find that deletion of Atg1 fails to rescue Sae2 from the degradation induced by CHX (Figure 5D).

Treatment of Dna2 with 100 μg/ml CHX after HO induction led to a modest decrease in Dna2 levels (Supplementary Figure S6A). Interestingly, the higher migrating band seen after DNA damage was lost after CHX treatment. Unlike Sae2, treatment of cycling cells or G2/M arrested cells with 20 mM caffeine did not result in decrease in Dna2 levels (Supplementary Figure S6B and S6C). These results suggest that DNA damage leads to the susceptibility of Dna2 for degradation following caffeine and CHX treatments.

Last we assessed whether CHX and caffeine treatment could lead to loss of Sae2 even after the protein had accumulated significantly after DNA damage. We arrested cells in G2/M with nocodazole, induced HO to create a single unrepaired DSB and treated with either CHX or caffeine 6 h after induction. There was a marked increase in the abundance of Sae2 after DSB induction and the protein remained stable for at least 9 h after DSB induction (Figure 5E). Caffeine or CHX addition resulted in degradation of Sae2 even when the treatment took place 6 h after damage, when the DNA damage checkpoint is fully activated (Figure 5F and G). Treatment with either CHX or caffeine at 6 h led to the appearance of lower migrating bands on the gel. These appear to be degradation intermediates. Taken together, these data show that Sae2 is an intrinsically unstable protein, and that this instability is not a result of post-translational modifications that occur following DNA damage.

### Caffeine treatment impairs resection and reduces RPA and Rad51 foci in mammalian cells

In mammalian cells, X-irradiation results in formation of Rad51 foci at sites of DNA damage. When Chinese Hamster Ovary (CHO) cells are treated with caffeine prior to exposure to irradiation, Rad51 foci are greatly diminished (52). While this observation may suggest that Rad51 itself is sensitive to caffeine, it may also be a result of impaired resection in the presence of caffeine.

**Figure 6.**
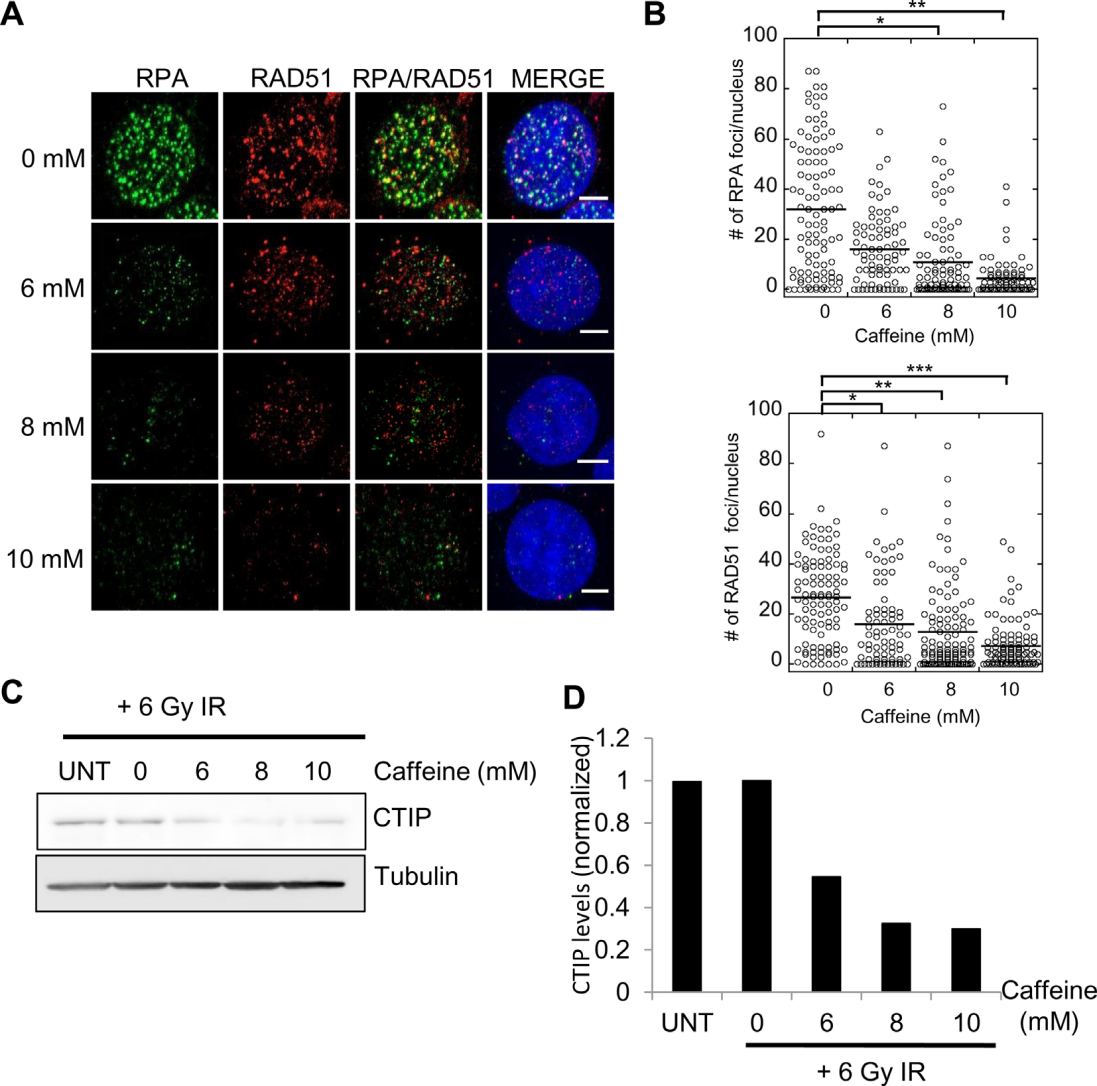
Caffeine treatment impairs RAD51 and RPA focus formation in HeLa cells. **(A)** Caffeine treatment impairs resection in HeLA cells. Increasing concentrations of caffeine added to HeLa cells 1 h prior to irradiation (6 Gy). Cells fixed 4 h after irradiation and immunostained with α-Rad51 or α-RPA antibodies. Decrease in RPA and Rad51 foci observed in caffeine-treated cells. **(B)** Quantification of foci. Brackets show comparisons with the untreated control that revealed statistically significant differences. * *P*-value<0.05, ** *P*-value<0.005, *** *P*-value<0.05, Wilcoxon rank sum test. **(C)** CtIP steady-state levels in irradiated HeLa cells following caffeine treatment. **(D)** Quantification of CtIP levels in irradiated HeLa cells following caffeine treatment. Graph depicts average of two independent experiments. Levels are depicted relative to the IR sample without caffeine.

To test if caffeine impairs resection in mammalian cells, we treated HeLa cells with different concentrations of caffeine 1 h prior to irradiation, allowed the cells to recover for 4 h, fixed the cells and immunostained with Rad51 and RPA antibodies. As previously reported (52), the number of Rad51 foci decreased in caffeine-treated cells. We found this decrease to be dose-dependent (Figure 6A and B). However, this reduction was not specific to Rad51, as the number of RPA foci also decreased in a dose-dependent manner. For example, cells treated with 10 mM caffeine resulted in a 3-fold and 7-fold decrease in Rad51 and RPA focus counts, respectively. Furthermore, the intensity of the Rad51 and RPA foci in caffeine-treated cells was weaker than the untreated controls. Given the high level of specificity of RPA for ssDNA tracts, the reduction in staining intensity is very likely to reflect a reduction in the average length of ssDNA tracts in these cells. Taken together with the more direct analysis of ssDNA tract lengths in yeast, these observations suggest that caffeine impairs resection in mammalian cells.

Next we determined if levels of CtIP, the human homolog of Sae2 (85), were reduced by caffeine treatment. We observed a 2- to 3-fold decrease in the steady-state levels of CtIP in irradiated cells after treatment with increasing amounts of caffeine (Figure 6C and D), similar to the effect of caffeine on Sae2 in budding yeast. These observations suggest that the mechanism through which caffeine limits DNA resection is substantially conserved between budding yeast and human cells.

## DISCUSSION

The mechanism by which cells sense DNA damage is of great interest and has important implications for cancer research. γ-H2AX has been proposed to play a role in the maintenance of the DNA damage checkpoint. Here we show that the removal of this modification after DSB repair is locus autonomous. This finding demonstrates that the sensing mechanism for the persistence of a DSB is local and not global, and may suggest that the global DNA damage checkpoint is turned on and off by coordinating many local sensing events.

Caffeine's inhibitory effect on the DNA damage checkpoint through the inhibition of ATR and ATM has long been established, though the mechanism of this inhibition remains poorly understood. Evidence from mammalian cells suggests that, apart from inhibiting the DNA damage checkpoint, caffeine treatment also inhibits HR (51,52). Caffeine was also shown to inhibit gene targeting, which requires much of the HR machinery (53). Here we show that caffeine targets one of the earliest processes of HR: 5′ to 3′ resection. Impairment of resection is particularly important as all HR processes depend on resection to create recombinogenic 3′-ended ssDNA. We note that caffeine treatment at lower concentrations (10 and 20 mM) did not completely inhibit SSA. Repair by SSA requires nonhomologous tail clipping that is dependent on Mec1 or Tel1 phosphorylation of Slx4 (86). Caffeine, therefore, affects SSA by two mechanisms; impairing resection and inhibiting nonhomologous tail clipping. It is possible that at lower caffeine concentrations, resection is attenuated but residual Mec1 or Tel1 activity allows nonhomologous tail clipping, therefore SSA is impaired but not abolished. At higher concentrations, Mec1 and Tel1 may be completely inhibited so repair is prevented by both inhibition of tail clipping and attenuation of resection. Yet another possibility is that the slower resection seen after 50 mM caffeine treatment (2.4 kb/h at 50 mM caffeine, 2.7 kb/h at 10 mM and 20 mM caffeine) leads to a more significant inhibition of repair at the higher concentrations.

In yeast, caffeine treatment affects enzymes involved both in the initiation of resection and extended resection. Sae2 and its mammalian homolog CtIP together with the MRX complex (MRN in mammalian cells) initiate resection after a DSB (5,85). Cells lacking Sae2 are impaired in resection (8). Here we show that caffeine treatment reduces levels of Sae2. We show that the turnover of Sae2 is very rapid, as inhibiting protein synthesis leads to a complete loss of Sae2 as soon as 30 min after treatment. Surprisingly, caffeine treatment 0.5 h after HO induction did not result in substantial impairment in the resection of the first 700 bp. This result may indicate that Sae2's role in initiation of resection is achieved by that time, and also explains the inability of caffeine treatment to increase NHEJ efficiency. Caffeine also acts as a TORC1 inhibitor, which regulates protein synthesis. Although Robert *et. al.* showed that Sae2 is degraded by autophagy following VPA treatment, we could not replicate this result. Further we find that abrogation of autophagy by deleting *ATG1* failed to rescue Sae2 from degradation following caffeine, rapamycin or CHX treatments. Moreover, the induction of autophagy by Atg13–8SA did not lead to the mislocalization of Sae2. Last, we show that inhibition of proteasomal degradation by PS-341 treatment stabilizes Sae2 levels in cells and prevents the reduction of resection following caffeine treatment. Because CHX led to rapid loss of Sae2 in WT cells both with and without DNA damage, and in *atg1*Δ cells, we propose that Sae2 has a short half-life independent of DNA damage or chemical treatment.

Extensive resection takes place through the Exo1 and Dna2/Sgs1-Top3-Rmi1 pathways. Exo1 levels are not significantly affected by caffeine treatment; however, Dna2 levels decrease in response to caffeine treatment. Interestingly, the rate of resection measured after caffeine treatment (2.4–2.7 kb/h in 10–50 mM caffeine) resembles the rate of resection measured in *sgs1*Δ cells (3 kb/h) (36). We conclude that caffeine affects resection through the damage-dependent degradation of Dna2 and the rapid turnover of Sae2.

We note that caffeine treatment leads to a more rapid loss of the DNA damage dependent phosphorylated form of Dna2 than the unmodified form. It is also possible that the maintenance of the phosphorylated state requires continuous Mec1 and Tel1 activity. In this case, the phosphorylated form of Dna2 would be dephosphorylated by caffeine treatment, leading indirectly to its degradation. Reduction in proteasomal activity by deletion of the transcription factor *RPN4* was sufficient to partially stabilize Dna2 levels after caffeine treatment.

Caffeine treatment sensitizes mammalian cells to irradiation and other DNA damaging agents. Caffeine was thought to do so through the inhibition of p53, ATM and ATR, although p53-proficient cancer cells are not radiosensitized by caffeine, suggesting that the main pathway by which caffeine acts is inhibition of processes that occur after the cells pass ‘start’ (48). Caffeine-induced radiosensitivity likely results from inhibition of HR because this radiosensitivity is not observed in HR-defective cells (50,51). Recently caffeine was shown *in vivo* to prevent gene targeting in mammalian cells independently of the DNA damage checkpoint (53). *In vitro*, caffeine treatment was shown in the same study to interfere with Rad51-mediated joint molecule formation. In an accompanying paper we corroborate these findings, and show that at higher concentrations caffeine fully displaces Rad51 filaments from the ssDNA. However, the findings presented here show that caffeine treatment targets a key step preceding Rad51 filament formation and homology searching. Our studies suggest that the effect of caffeine on resection is conserved from yeast to mammals. The data presented here further our mechanistic understanding of caffeine-mediated radiosensitivity, informing the ongoing effort to develop DNA repair inhibitors for cancer treatment (87–90).

## SUPPLEMENTARY DATA

Supplementary Data are available at NAR Online.

SUPPLEMENTARY DATA
