# Supplementary material for: Caffeine impairs resection during DNA break repair by reducing the levels of nucleases Sae2 and Dna2
Source: Nucleic Acids Res. 2015 May 27;43(14):6889–901. doi: 10.1093/nar/gkv520 (PMC4538808; doi:10.1093/nar/gkv520)
Supplement: SUPPLEMENTARY DATA [file supp_43_14_6889__index.html]

Caffeine impairs resection during DNA break repair by reducing the levels of nucleases Sae2 and Dna2 — Caffeine impairs resection during DNA break repair by reducing the levels of nucleases Sae2 and Dna2 — Caffeine impairs resection during DNA break repair by reducing the levels of nucleases Sae2 and Dna2 — SUPPLEMENTARY DATA 

# Caffeine impairs resection during DNA break repair by reducing the levels of nucleases Sae2 and Dna2

## SUPPLEMENTARY DATA

- SUPPLEMENTARY DATA
