# Supplementary material for: Caffeine impairs resection during DNA break repair by reducing the levels of nucleases Sae2 and Dna2
Source: Nucleic Acids Res. 2015 May 27;43(14):6889–901. doi: 10.1093/nar/gkv520 (PMC4538808; doi:10.1093/nar/gkv520)
Supplement: SUPPLEMENTARY DATA [file supp_gkv520_nar-03479-d-2014-File008.pdf]

Figure S1

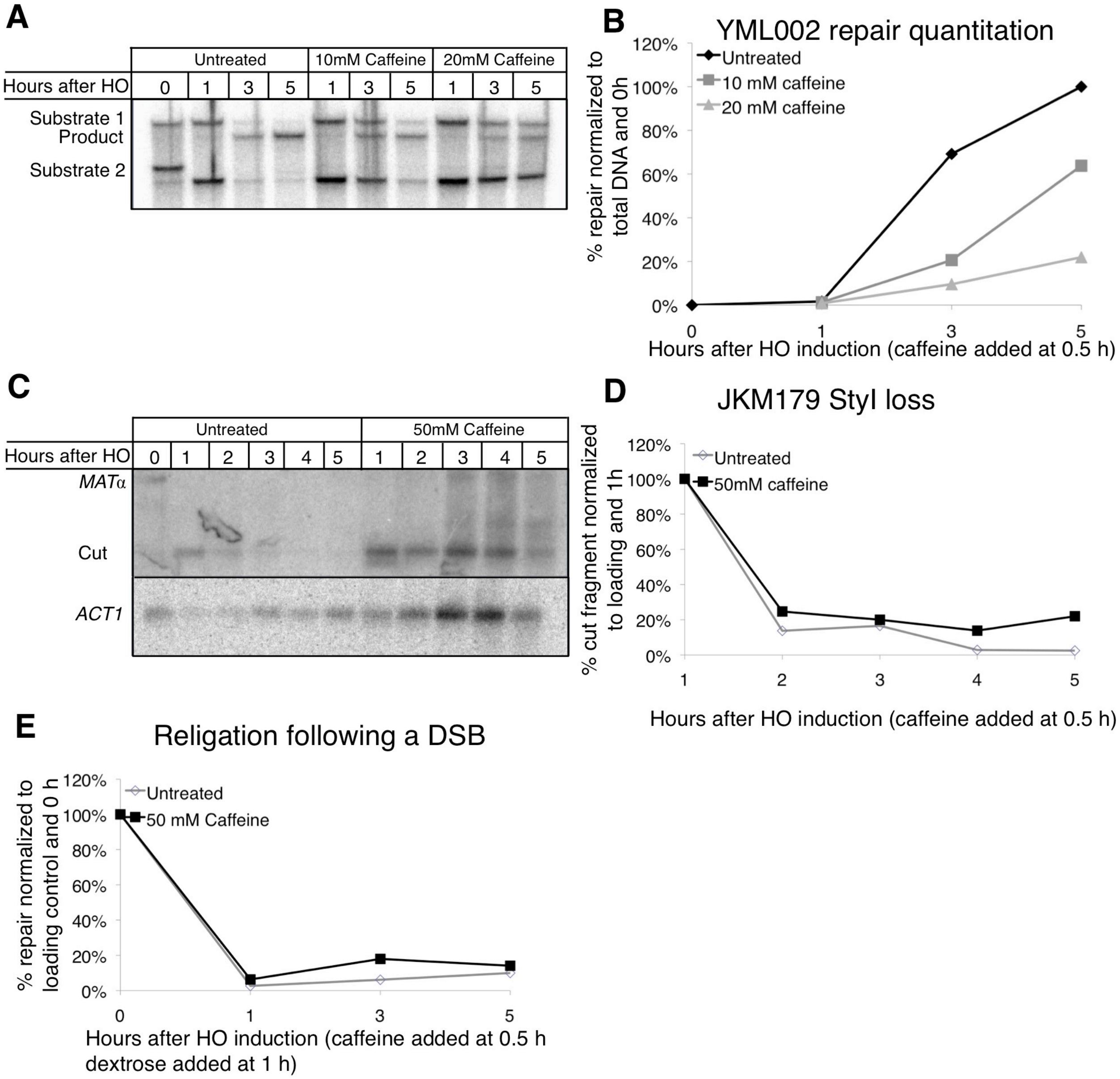

Supplemental Figure 1 – 10 mM and 20 mM Caffeine treatment impairs SSA

A) Southern blot to assess SSA after 10 mM and 20 mM caffeine treatment.

B) Quantification of product formation. Product was normalized to total DNA and 0 h.

C) Southern blot to detect the loss of a 700 bp cut fragment following DSB. HO was induced in JKM179. Culture was split at 0.5 h and half was treated with 50 mM caffeine. Genomic DNA was digested with StyI and probed with a *MAT* distal probe.

D) Quantification of the cut fragment loss. Signal was normalized to loading control (*ACT1* probe) and to 1 h signal.

E) Religation experiment. HO was induced in JKM179. 0.5 h following induction the culture was treated with 50 mM caffeine. 1 h after induction a final concentration of 2% dextrose was added to the culture. Signal was normalized to a loading control and to 1 h signal.

Figure S2

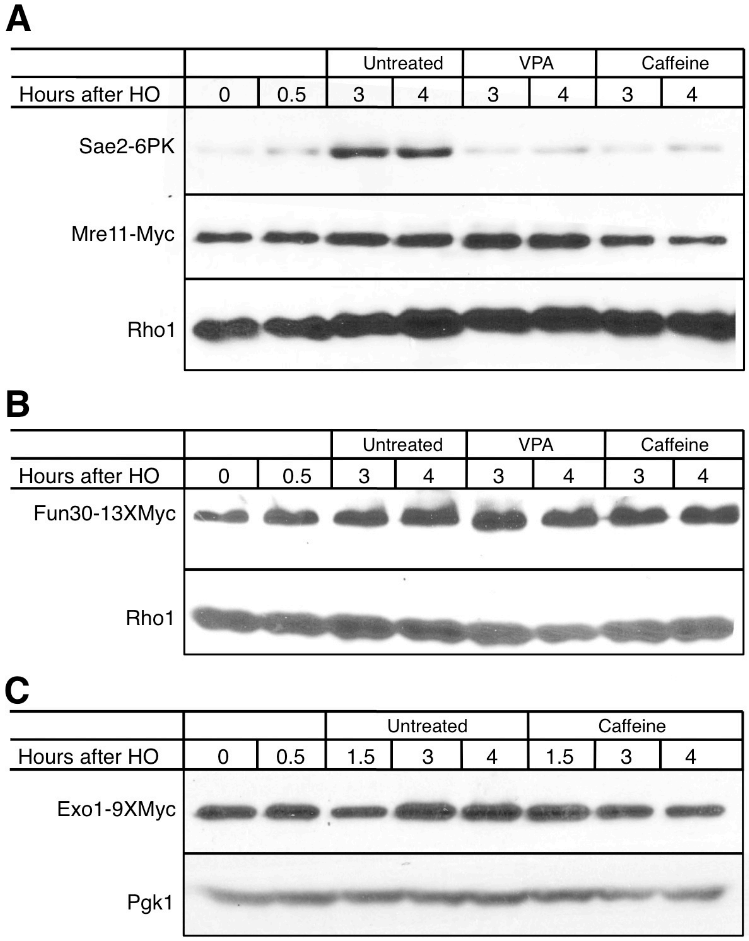

Supplemental Figure 2 – Effect of caffeine and VPA on resection enzymes

A) Caffeine leads to degradation of Sae2 but not Mre11. Cells were arrested in nocodazole prior to HO induction, and treated with either VPA or 20 mM caffeine 0.5 h following HO induction.

B) Caffeine and VPA do not lead degradation of Fun30. Cells were arrested in nocodazole prior to HO induction, and treated with either VPA or 20 mM caffeine 0.5 h following HO induction.

C) Caffeine does not lead to degradation of Exo1. Cells were arrested in nocodazole prior to HO induction, and treated with 20 mM caffeine 0.5 h following HO induction.

Figure S3

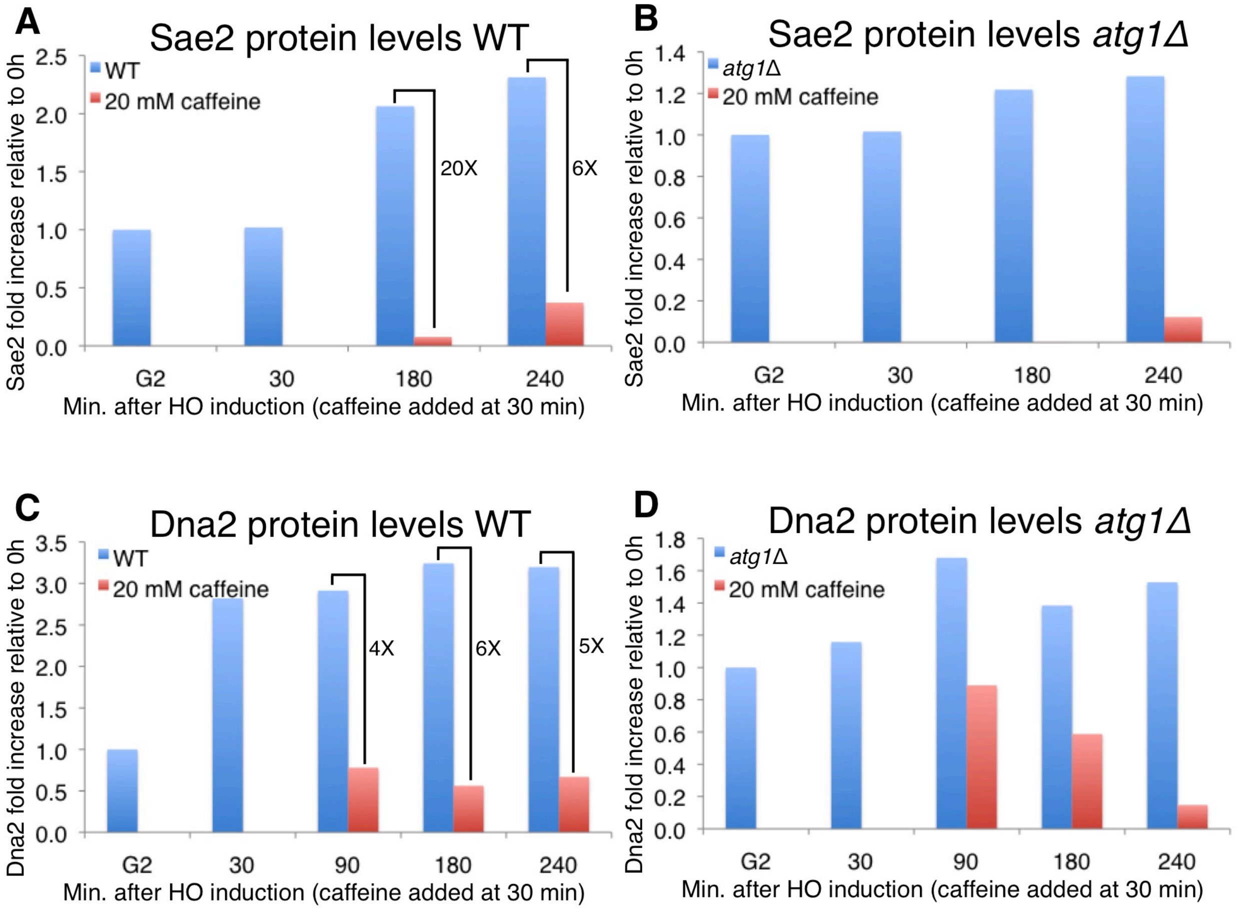

Supplemental Figure 3 – Western blot quantification. All quantifications were done using “Quantity One” software.

A) Quantification of Sae2 WT western blot. Sae2 signal from lanes 1-6 from figure 3A was normalized to loading control and to 0 h (G2 – Lane 1).

B) Quantification of Sae2 *atg1*Δ western blot. Sae2 signal from lanes 7-12 from figure 3A was normalized to loading control and to 0 h (G2 – Lane 7).

C) Quantification of Dna2 WT western blot. Dna2 signal from figure 3B was normalized to loading control and to 0 h (G2).

D) Quantification of Dna2 *atg1*Δ western blot. Dna2 signal from figure 3C was normalized to loading control and to 0 h (G2).

Figure S4

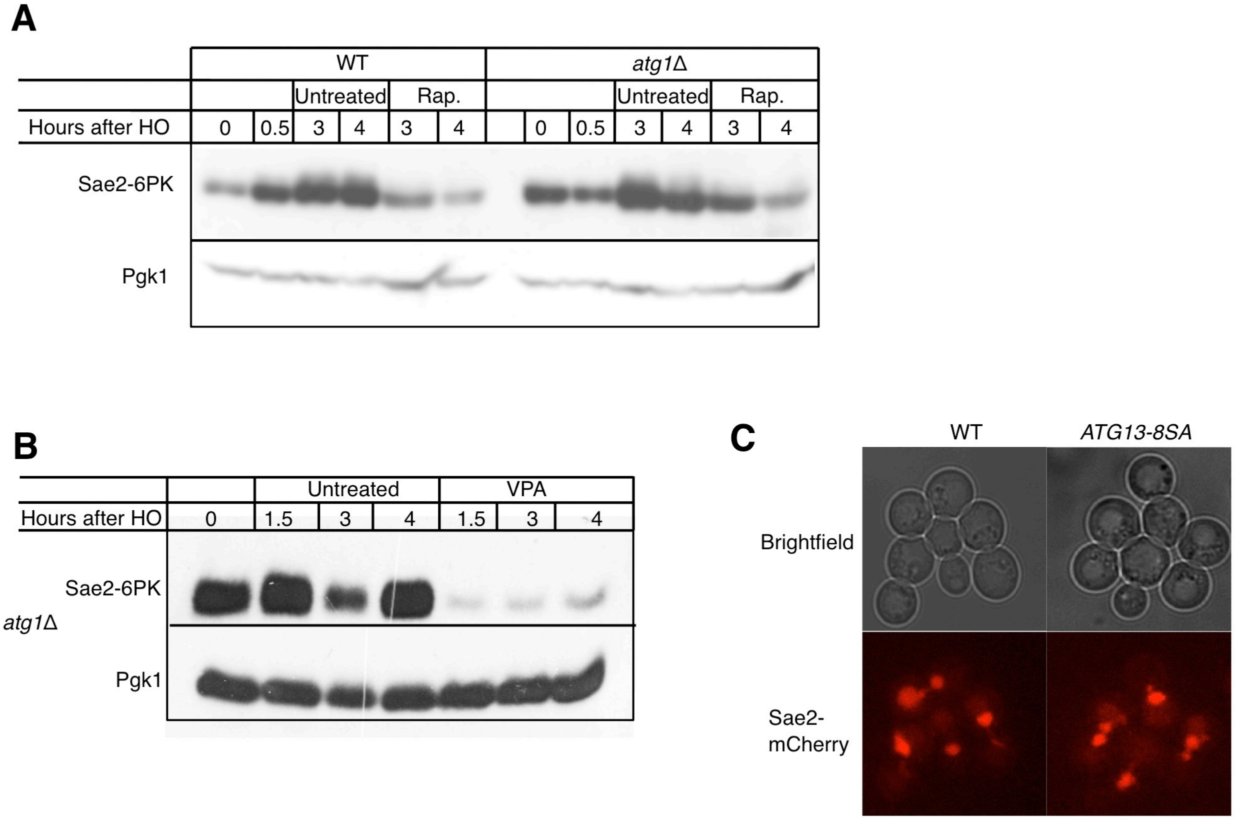

Supplemental Figure 4 – Sae2 is degraded after valproic acid (VPA) treatment independent of autophagy

A) Western blot of the effect of rapamycin treatment on Sae2. 200 ng/ml rapamycin was added to the medium 0.5 h after HO induction in both WT and *atg1*Δ cells.

B) Western blot analysis of the effect of VPA treatment on Sae2. 10 mM VPA was added to the media 0.5 h after HO induction in *atg1*Δ cells.

C) Sae2 is not mislocalized following induction of autophagy by Atg13-8SA overexpression. Sae2-mCherry was visualized in WT and Atg13-8SA overexpressing cells 6 h following HO induction.

Figure S5

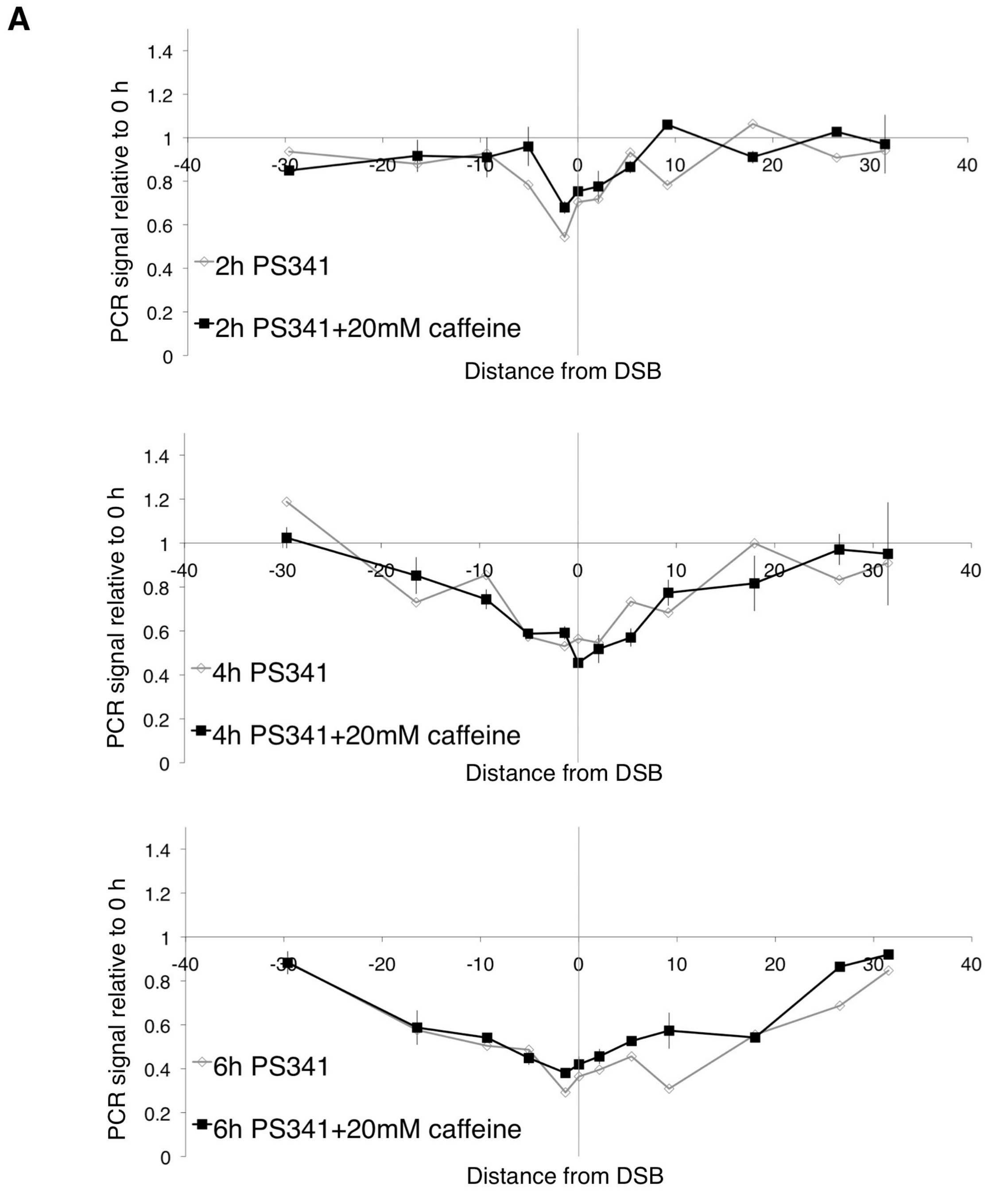

Supplemental Figure 5 - inhibition of proteasomal degradation abrogates the impairment of resection following caffeine treatment.

A) Resection in *rpn4*Δ *pdr5*Δ strain without caffeine treatment (gray diamonds), or when 20 mM caffeine (black squares) was added 0.5 h after HO induction. Cells were arrested in G2/M by nocodazole treatment for 3 h. At 0 h HO was induced and cells were treated with 100 mM PS-341. Error bars represent ranges.

Figure S6

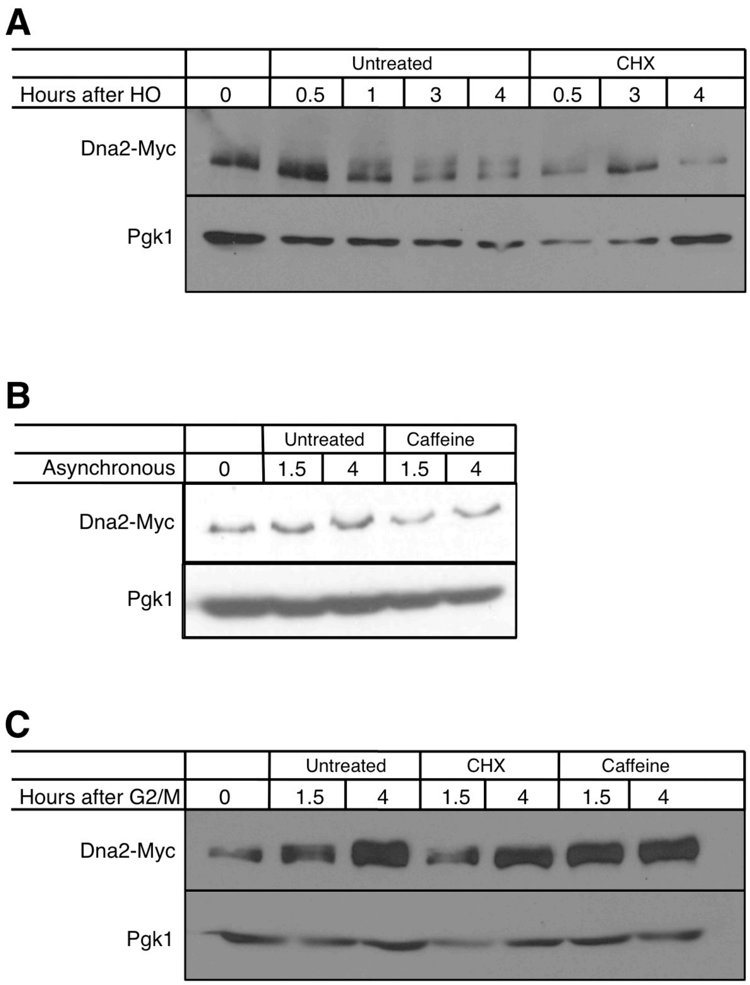

Supplemental Figure 6 – Dna2 is not rapidly degraded following inhibition of protein synthesis.

A) Dna2 levels decrease following CHX treatment. HO was induced in G2/M arrested cells. Cells were treated with 100 µg/ml CHX 0.5 h following HO induction.

B) Dna2 levels did not decrease following caffeine treatment in cells that did not suffer a DSB. 20 mM caffeine was added to an asynchronous culture and Dna2 levels were monitored 1.5 h and 4 h following treatment.

C) Dna2 is not degraded following caffeine or CHX treatment in G2/M cells. Cells were arrested in G2/M with nocodazole. 20 mM caffeine or 100 µg/ml CHX were added to the culture. Dna2 levels were monitored 1.5 h and 4 h following treatment.

Table I – Strain list

Strain name	Genotype
JKM179	*hoΔ hml::ADE1 MATα hmr::ADE1 ade1-110 leu2,3-112 lys5 trp1::hisG ura3-52 ade3::GAL:HO*
JKM139	*JKM179 isogenic, MATa*
YML002	*JKM139 (HO cut site deleted) Cen3HOcs::HPH 2 kb homology to the left of the HOcs inserted to the right of Cen3, 97700-97800 Ch6::HOcs-NAT*
tGI354	*JKM139 MATa-inc (+CA), arg5,6::MATa-HPH*
R1304(Ira Lab)	*JKM139 DNA2-9xMYC::TRP1*
R1306(Ira Lab)	*JKM139 Fun30-3x-FLAG::KAN EXO1-9x MYC::TRP1*
R1307	*JKM179 Fun30-13x Myc::NAT RFA1-3X FLAG::KAN*
R1206	*JKM139 Mre11-Myc::KAN SAE2-6PK::TRP1 Exo1-Flag::KANMX*
CY9666	*JKM139, Sae2-6PK, atg1::KAN*
VE18	*JKM179, GFP-ATG8 URA3 (pRS316)*
VE150	*R1304 atg1::URA3*
VE114	*JKM179 GFP-Atg8 URA3 (pRS316) atg1::KAN*
sDW133	*JKM179 pdr5::HPH rpn4::NAT SAE2-6PK::TRP1*
sDW134	*JKM179 pdr5::HPH rpn4::NAT DNA2-9xMYC::TRP1*
